# Supplementary material for: Optimization of differentiation and transcriptomic profile of THP-1 cells into macrophage by PMA
Source: PLoS One. 2023 Jul 17;18(7):e0286056. doi: 10.1371/journal.pone.0286056 (PMC10351730; doi:10.1371/journal.pone.0286056)
Supplement: S1 File — (ZIP) [file pone.0286056.s001.zip › supporting files/RNA-seq report.zip!/RNA-seq report/4_GOEnrichment_result/induce_vs_control/DAG/induce_vs_control.DEG_bp_DAG.pdf]

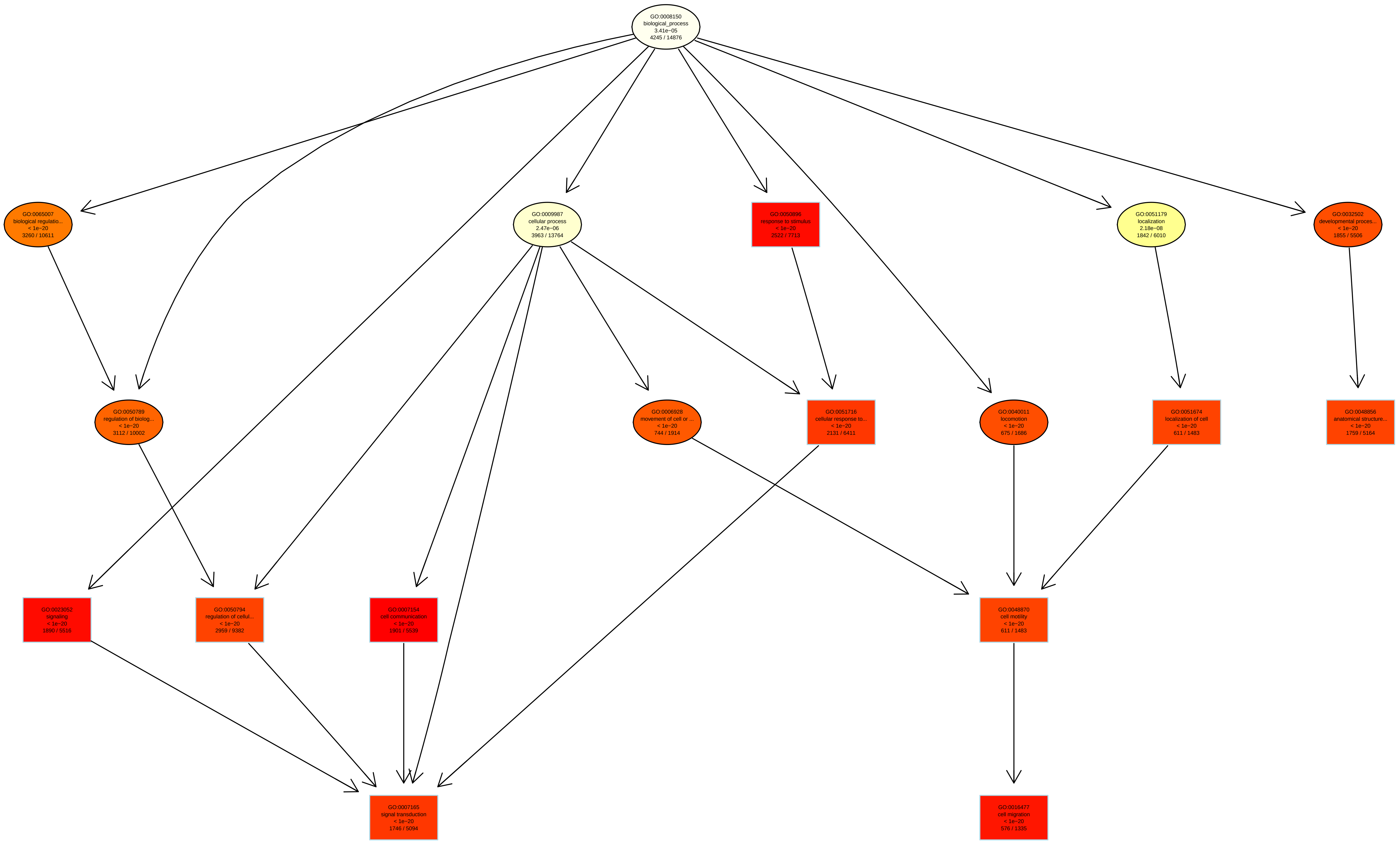
GO:0008150
biological_process
3.41e−05
4245 / 14876
GO:0065007
biological regulatio...
< 1e−20
3260 / 10611
GO:0009987
cellular process
2.47e−06
3963 / 13764
GO:0050896
response to stimulus
< 1e−20
2522 / 7713
GO:0051179
localization
2.18e−08
1842 / 6010
GO:0032502
developmental proces...
< 1e−20
1855 / 5506
GO:0050789
regulation of biolog...
< 1e−20
3112 / 10002
GO:0006928
movement of cell or ...
< 1e−20
744 / 1914
GO:0051716
cellular response to...
< 1e−20
2131 / 6411
GO:0040011
locomotion
< 1e−20
675 / 1686
GO:0051674
localization of cell
< 1e−20
611 / 1483
GO:0048856
anatomical structure...
< 1e−20
1759 / 5164
GO:0023052
signaling
< 1e−20
1890 / 5516
GO:0050794
regulation of cellul...
< 1e−20
2959 / 9382
GO:0007154
cell communication
< 1e−20
1901 / 5539
GO:0048870
cell motility
< 1e−20
611 / 1483
GO:0007165
signal transduction
< 1e−20
1746 / 5094
GO:0016477
cell migration
< 1e−20
576 / 1335
